# Molecular prevalence of *Bartonella*, *Babesia*, and hemotropic *Mycoplasma* species in dogs with hemangiosarcoma from across the United States

**DOI:** 10.1371/journal.pone.0227234

**Published:** 2020-01-10

**Authors:** Erin Lashnits, Pradeep Neupane, Julie M. Bradley, Toni Richardson, Rachael Thomas, Keith E. Linder, Matthew Breen, Ricardo G. Maggi, Edward B. Breitschwerdt

**Affiliations:** 1 Intracellular Pathogens Research Laboratory, Comparative Medicine Institute, College of Veterinary Medicine, North Carolina State University, Raleigh, North Carolina, United States of America; 2 Department of Molecular Biomedical Sciences, Comparative Genomics, College of Veterinary Medicine, North Carolina State University, Raleigh, North Carolina, United States of America; 3 Department of Population Health and Pathobiology, College of Veterinary Medicine, North Carolina State University, Raleigh, North Carolina, United States of America; 4 Department of Clinical Sciences, College of Veterinary Medicine, North Carolina State University, Raleigh, North Carolina, United States of America; Colorado State University, UNITED STATES

## Abstract

Hemangiosarcoma (HSA), a locally invasive and highly metastatic endothelial cell neoplasm, accounts for two-thirds of all cardiac and splenic neoplasms in dogs. *Bartonella* spp. infection has been reported in association with neoplastic and non-neoplastic vasoproliferative lesions in animals and humans. The objective of this study was to determine the prevalence of *Bartonella* spp. in conjunction with two other hemotropic pathogens, *Babesia* spp. and hemotropic *Mycoplasma* spp., in tissues and blood samples from 110 dogs with histopathologically diagnosed HSA from throughout the United States. This was a retrospective, observational study using clinical specimens from 110 dogs with HSA banked by the biospecimen repository of the Canine Comparative Oncology and Genomics Consortium. Samples provided for this study from each dog included: fresh frozen HSA tumor tissue (available from n = 100 of the 110 dogs), fresh frozen non-tumor tissue (n = 104), and whole blood and serum samples (n = 108 and 107 respectively). Blood and tissues were tested by qPCR for *Bartonella*, hemotropic *Mycoplasma*, and *Babesia* spp. DNA; serum was tested for *Bartonella* spp. antibodies. *Bartonella* spp. DNA was amplified and sequenced from 73% of dogs with HSA (80/110). In contrast, hemotropic *Mycoplasma* spp. DNA was amplified from a significantly smaller proportion (5%, p<0.0001) and *Babesia* spp. DNA was not amplified from any dog. Of the 100 HSA tumor samples submitted, 34% were *Bartonella* PCR positive (32% of splenic tumors, 57% of cardiac tumors, and 17% of other tumor locations). Of 104 non-tumor tissues, 63% were *Bartonella* PCR positive (56% of spleen samples, 93% of cardiac samples, and 63% of skin/subcutaneous samples). Of dogs with *Bartonella* positive HSA tumor, 76% were also positive in non-tumor tissue. *Bartonella* spp. DNA was not PCR amplified from whole blood. This study documented a high prevalence of *Bartonella* spp. DNA in dogs with HSA from geographically diverse regions of the United States. While 73% of all tissue samples from these dogs were PCR positive for *Bartonella* DNA, none of the blood samples were, indicating that whole blood samples do not reflect tissue presence of this pathogen. Future studies are needed to further investigate the role of *Bartonella* spp. in the development of HSA.

## Introduction

There are clear precedents for the involvement of bacterial infection in neoplastic development. Within the past 25 years, a considerable volume of research has been conducted on the oncogenic properties of infectious agents such as bacteria, mycoplasma, protozoa, and viruses.[1,2] Currently, infectious agents are accepted as a cause or co-factor in anywhere from 5–50% of human cancers worldwide, depending on the geographic region and its development status.[1–3] The involvement of infectious agents in the pathogenesis of some human cancers is therefore well established. The majority of infectious agents implicated in oncogenesis are viruses, such as Epstein Barr virus, human papillomaviruses, and Kaposi’s sarcoma-associated herpesvirus.[1] These viruses have direct oncogenic properties through integration of viral genomes into host cells, or by secretion of gene products into healthy cells to create tumor cells. The extent to which other infectious agents, such as bacteria, lack the inherent oncogenic properties of their viral counterparts remains unclear. Bacteria most often promote cancer development indirectly through persistent replication, inflammation and chronic tissue damage.[4,5] *Helicobacter pylori*, for example, colonizes and replicates within the gastric mucosa, resulting in a chronic pro-inflammatory response that promotes cancer risk and oncogenesis of gastric cancer by altering epithelial cell proliferation and apoptosis.[6] With the difficulty of assessing causality, particularly for certain rare cancer types, there may be roles for other pathogenic bacteria in a range of different cancers that have not yet been discovered.[7]

**Fig 1:**
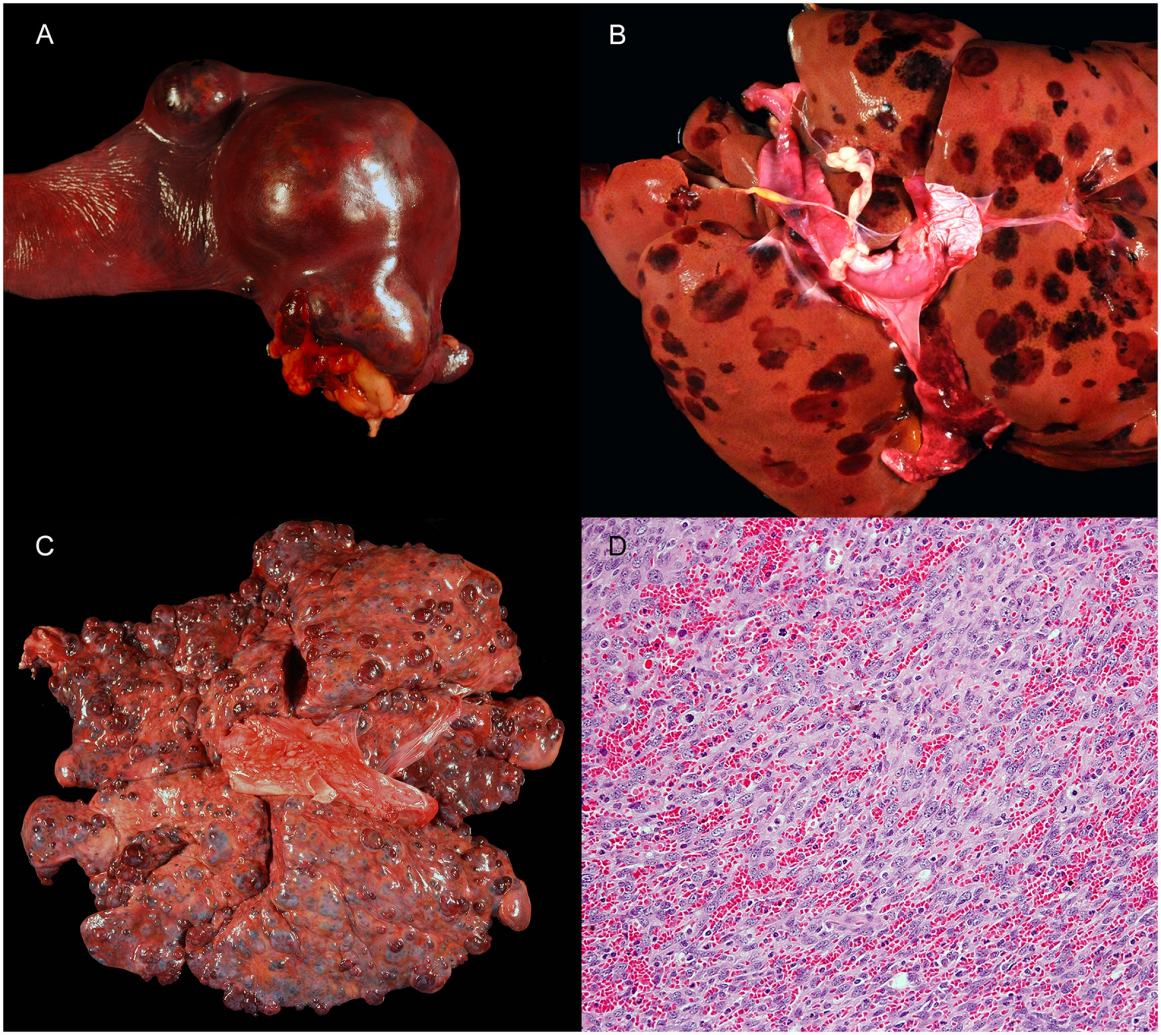
Images of hemangiosarcoma (HSA) tumors from dogs. A) Primary HSA mass in a spleen. B) Metastatic HSA in a liver with multifocal masses in all lobes. C) Metastatic hemangiosarcoma in the lungs with multifocal masses in all lobes. D) Photomicrograph of a splenic HSA from a dog in the current study that was PCR positive for *B*. *henselae*. 20X magnification. Hematoxylin and eosin. Credits: Talley A (image A), Sommer S (image B), Rasche B (image C) and Barnes J (image D).

Hemangiosarcoma (HSA) is a highly aggressive endothelial cell cancer that is associated with local invasiveness and a high metastatic potential in dogs (Fig 1).[8,9] The most common neoplasm of the spleen,[10] this cancer is found in up to 2% of all dogs with tissues submitted for autopsy.[11] While HSA may affect any dog breed, several of the most popular family owned pure breeds are highly predisposed, including the golden retriever, Labrador retriever and German Shepherd Dog.[12–14] Splenic HSA is among the most common canine cancers encountered in clinical practice, accounting for approximately two thirds of all splenic tumors (neoplasms of the spleen, benign or malignant, comprise approximately half of all splenic pathology in dogs).[9] Cardiac HSA is less common than splenic HSA (most studies reporting less than 1% of all canine neoplasms), but remains the most common cardiac tumor of dogs.[15,16] Other primary tumor locations such as the liver and skin are reported, but rare.

Splenic HSA is often difficult to diagnose without resorting to splenectomy, a major invasive abdominal surgery.[8,17] Moreover, as an indolent disease, malignant HSA masses that develop within the abdominal cavity often remain undetected until reaching an advanced stage, at which time there is a high risk of spontaneous rupture potentially leading to untreatable and ultimately fatal internal hemorrhage.[18] Because of this risk, as well as the lack of broadly effective chemotherapeutics, the prognosis is poor. The median survival after diagnosis ranges from less than 3 weeks with splenectomy surgery alone to six months with surgery plus cancer chemotherapy.[19,20] As a result there is a critical need for improved diagnostic modalities for earlier detection of HSA, as well as new treatments and preventative strategies to improve outcomes for this common tumor of family pets.

Despite substantial research, the etiology and pathogenesis of canine HSA remains unclear. As a malignant tumor of vascular endothelial cells, factors that have been hypothesized to contribute to the pathogenesis of HSA include chronic inflammation, macrophage activation, hypoxia, and angiogenesis.[21] *In vitro*, HSA cells produce growth factors promoting angiogenesis, including vascular endothelial growth factor-A (VEGF-A), platelet-derived growth factor-β (PDGF-β), and basic fibroblast growth factor (bFGF), and genes involved in inflammation, angiogenesis, and cellular adhesion and invasion can distinguish HSA cells from non-malignant endothelial cells.[9,21]

Because of established links between chronic intracellular infections, inflammation, and angiogenesis, efforts are being made to determine if chronic intravascular infection with bacteria or protozoa could contribute to HSA development in dogs. One previous study from our laboratory examined the molecular prevalence of blood (erythrocyte)-borne pathogens in a small cohort of dogs from the southeastern United States with and without splenic pathology.[22] We found that *Bartonella* spp. were significantly more common than *Babesia* spp. or hemotropic *Mycoplasma* spp. in formalin-fixed, paraffin embedded biopsy samples from splenic HSA: 26% of dogs were positive for *Bartonella* spp. compared to 2% for *Babesia* spp. (p < 0.001) and 6% for hemotropic *Mycoplasma* spp. (p = 0.006). Moreover, *Bartonella* spp. were found more often in splenic HSA biopsy samples compared to samples from a non-neoplastic inflammatory disorder of the spleen (lymphoid nodular hyperplasia, LNH) and histologically normal splenic tissue from specific-pathogen-free dogs.[22] We have subsequently documented that *Bartonella* spp. DNA can be amplified from angioproliferative lesions in cats, cows, dogs and horses.[23] In addition, it has been demonstrated that multiple *Bartonella* spp. (*B*. *bacilliformis*, *B*. *quintana*, *B*. *henselae*, and three *Bartonella vinsonii* subsp. *berkhoffii* genotypes) can induce the *in vitro* production of VEGF.[23–25] *Bartonella* spp. can cause endothelial proliferative disorders, including bacillary angiomatosis and peliosis hepatis, in dogs and humans.[26–31] In combination, these observations suggest the potential for involvement of intra-erythrocytic and endotheliotropic *Bartonella* spp. in the initiation and/or progression of vascular endothelial neoplasia in dogs.

However, in our previous case control study demonstrating an association between *Bartonella* spp. infection and HSA,[22] samples were restricted to a single geographical region (North Carolina) and a single anatomical site (splenic HSA). Seroprevalence studies show that *Bartonella* spp. exposure in dogs can be seen throughout the United States, and there are relatively small but statistically significant regional differences in seroprevalence.[32,33] Additionally, the presence of *Bartonella* spp. DNA in splenic tissue could potentially by explained by the spleen’s role in removal of hemotropic parasites from systemic circulation, or by *Bartonella* spp. bacteremia at the time of splenic specimen collection. To address these outstanding questions, this study sought to further clarify the potential involvement of *Bartonella* spp. in HSA in a broader anatomic and geographic context.

The objective of this study was to determine the prevalence of *Bartonella* spp. in conjunction with two other hemotropic pathogens, *Babesia* spp. and hemotropic *Mycoplasma* spp., in tissues and blood samples from dogs with histopathologically diagnosed HSA from throughout the United States. Our hypotheses were: 1) the prevalence of *Bartonella* spp. infection in dogs with HSA would be greater than the prevalence of *Babesia* or hemotropic *Mycoplasma* spp., and 2) the prevalence of *Bartonella* spp. infection in dogs with HSA in the spleen will be similar to prevalence in dogs with HSA in other anatomic locations, such as cardiac muscle.

## Methods

### Study design and sample sources

This was a retrospective, observational, descriptive study of 110 dogs with HSA. Specimens used for this study were previously collected and banked by the Canine Comparative Oncology and Genomics Consortium (CCOGC) based on previously published standard operating procedures.[34]. Briefly, the CCOGC collected samples from eight participating veterinary university teaching hospitals starting in 2006 with the goal of creating a repository of clinical samples from dogs with common naturally occurring cancers. Prior to sample submission to CCOGC, dogs were given a definitive diagnosis of neoplasia based on histopathology performed by a board-certified veterinary pathologist at the diagnostic laboratory of each participating university. Tissue samples for the CCOGC biospecimen repository were obtained from the primary tumor via surgical biopsy or post-mortem collection (HSA tumor tissue). As an internal control, tissue samples from each dog were also obtained from adjacent grossly normal tissue in the same organ, or if no grossly normal tissue in the affected organ was apparent, skin biopsies were obtained (non-tumor tissue). Tissue submission (HSA tumor and non-tumor) from each dog required provision of both formalin-fixed (subsequently stored in ethanol) and non-fixed specimens snap-frozen in liquid nitrogen (subsequently stored at -80° C). Dogs also had serum and whole blood collected at the time of tissue sampling for submission to the CCOGC.

For this study, samples from dogs diagnosed with HSA were provided by the CCOGC repository to the investigators. Four sample types were provided for pathogen testing: fresh frozen HSA tumor tissue, fresh frozen non-tumor tissue, whole blood, and serum. Two sample types were provided for histopathological confirmation of tissue type and tumor presence included: formalin-fixed HSA tumor tissue and formalin-fixed non-tumor tissue. Any dog that had a diagnosis of HSA (as determined by the CCOGC SOP), and had an adequate amount of fresh frozen tissue stored in the biorepository at the time of the investigators’ request for specimens, was included. This study, therefore, included 110 dogs with samples collected between May 2008 and November 2011.

Because of previous sample requests or lack of submission of all requested samples to the CCOGC, there were samples missing when provided to the investigators. Of the 110 dogs, 91 had a complete set of the 4 sample types for pathogen testing (fresh frozen HSA tumor tissue, fresh frozen non-tumor tissue, whole blood, serum) provided to the investigators. No dog was missing more than one sample type, and all dogs had at least one fresh-frozen tissue sample (HSA tumor, non-tumor tissue, or both) available for testing. For this reason, dogs with missing samples for pathogen testing were not excluded from the study. Similarly, of the 110 dogs only 37 had a complete set of the 2 sample types for histopathological confirmation (formalin-fixed HSA tumor tissue, formalin-fixed non-tumor tissue) provided to the investigators. Of the 110 dogs, 37 had formalin-fixed HSA tumor tissue and 93 had formalin-fixed non-tumor tissue provided. There were no formalin-fixed samples of HSA tumor or non-tumor tissue from cardiac tumors available for independent histopathologic review. Because a large proportion of dogs were missing formalin-fixed samples for independent histopathological confirmation, the subgroups of tissues with independent histopathologic confirmation of tissue type and tumor presence was analyzed separately.

The CCOGC provided demographic information for each dog, including age (years), breed, weight (kg) and sex and neuter status. The date and geographic location (university teaching hospital) of sample collection was also provided. The anatomic location of tumor and non-tumor tissue samples for each dog was also provided.

### Independent confirmation of demographic and histologic data

When possible, information provided by the CCOGC was independently confirmed by the investigators for this study. Histopathology reports providing the diagnosis of hemangiosarcoma were provided for 102 dogs; these were reviewed by one author (EL) to confirm hemangiosarcoma diagnosis and tumor location.

For dogs with formalin-fixed biopsy samples provided (see above), biopsies were independently evaluated by a board-certified veterinary pathologist (KL) to confirm the tissue and tumor origin of the biopsy. Formalin fixed tissues were submitted to the NCSU College of Veterinary Medicine histology laboratory (Raleigh NC) and tissues were embedded in paraffin blocks (FFPE blocks). Slides containing 5 um sections were prepared from the FFPE blocks and stained with hematoxylin and eosin (H&E). Samples were categorized by organ type and HSA tumor or non-tumor tissue based on H&E staining. If the tissue of origin was not able to be determined from the biopsy (5 tumor samples and 2 non-tumor tissues), or if no formalin-fixed sample was provided (68 tumor samples and 14 non-tumor tissues), the tissue of origin was categorized by the information provided by the CCOGC and the original diagnostic histopathology reports. Non-tumor tissues from skin or various subcutaneous tissues (including hair, skin, adipose, skeletal muscle, or mammary gland, or any combination of these tissues) were categorized as skin/SQ for analysis.

### Pathogen detection methods

DNA was extracted from EDTA anti-coagulated blood and fresh frozen tissue samples using a Qiagen DNeasy^®^ Blood and Tissue kit (Qiagen, Valencia, CA) following the manufacturer’s protocols. For each tissue sample (HSA tumor and non-tumor), a 25 mg piece of tissue was excised from the entire sample using a new, prepackaged sterile scalpel. DNA yield and quality was assessed by spectrophotometry (Nanodrop, Wilmington, DE).

Each DNA sample was screened for the presence of *Bartonella* spp. DNA using conventional and qPCR, and *Babesia* spp. and hemotropic *Mycoplasma* spp. using qPCR. *Bartonella* qPCR was performed using primers targeting the 16S-23S intragenic transcribed spacer (ITS) region of *Bartonella* species as described previously[35], in conjunction with a BsppITS438 FAM-labeled hydrolysis probe (TaqMan, Applied Biosystems, Foster City, CA, USA). *Bartonella* qPCR was performed at two dilutions for each sample (using 1 uL and 5 uL of template DNA respectively). PCR screening for *Babesia* spp. and hemotropic *Mycoplasma* spp. were carried out as described previously.[22] Briefly, oligonucleotides Myco16S-322s (5’ GCCCATATTCCTACGGGAAGCAGCAGT 3’) and Myco16S-938as (5’ CTCCACCACTTGTTCAGGTCCCCGTC 3’) were used as forward and reverse primers respectively for hemotropic *Mycoplasma* spp. DNA amplification. Oligonucleotides Piro18S-144s (5’ GATAACCGTGSTAATTSTAGGGCTAATACATG 3’) and Piroplasma18S-722as (5’ GAATGCCCCCAACCGTTCCTATTAAC 3’) were used as forward and reverse primers respectively for *Babesia* spp. DNA amplification.

Amplification was performed in a 25-μl final volume reaction containing 12.5 μl of MyTaq Premix (Bioline), 0.2 μl of 100 μM of each forward primer, reverse primer (IDT^®^ DNA Technology), 7.1 μl of molecular–grade water, and 5 μl of DNA from each sample tested. PCR negative controls were prepared using 5 μl of DNA from blood of a healthy dog. Positive controls for PCR were prepared by using 5 μl of DNA from previously characterized positive dog (clinical cases). Conventional PCR was performed in an Eppendorf Mastercycler EPgradient^®^ under the following conditions: a single hot-start cycle at 95°C for 2 minutes followed by 55 cycles of denaturing at 94°C for 15 seconds, annealing at 68°C for 15 seconds, and extension at 72°C for 18 seconds. Amplification was completed by an additional cycle at 72°C for 1 minute, and products were analyzed by 2% agarose gel electrophoresis with detection using ethidium bromide under ultraviolet light.

Validation of positive results was performed by Sanger sequencing of amplicons followed by chromatogram evaluation and sequence alignment using Contig-Express and Align X software (Vector NTI Suite 10.1, Invitrogen Corp, CA, USA). For bacterial species identification, DNA sequences were analyzed for nucleotide sequence homology at NCBI nucleotide database using BLAST version 2.0. A sample was considered *Bartonella* spp. PCR positive if one or more PCR tests (qPCR or conventional PCR) were positive (tests run in parallel). Stringent processing methods were used to avoid DNA carryover during tissue processing.[36] Specifically, tissue samples were processed independently using manual DNA extraction. For all batches of DNA extractions, between 2 and 4 blanks samples (water) were used as negative controls. All negative controls for DNA extractions rendered negative results on all PCR assays. DNA carryover after PCR amplification was avoided by processing each sample in three separate laboratory rooms (one for sample sorting and DNA extraction, a second for PCR processing, and a third for PCR analysis post amplification), and strict use of personal protective equipment for sample handling by laboratory personnel.

For IFA testing, *Bartonella* antibodies were determined using 3 cell culture grown *Bartonella* spp. (*Bartonella henselae*, *Bartonella vinsonii* subsp. *berkhoffii*, and *Bartonella koehlerae*) as antigens and following standard immunofluorescent antibody assay (IFA) techniques.[37,38] Briefly, bacterial colony isolates were passed from agar plate grown cultures into permissive cell lines. For each antigen, heavily infected cell cultures were spotted onto 30-well Teflon-coated slides (Cel-Line/Thermo Scientific), air-dried, acetone-fixed, and stored frozen. Fluorescein conjugated goat anti-dog IgG (KPL, SeraCare, Milford MA) was used to detect bacteria within cells using a fluorescent microscope (Carl Zeiss Microscopy, LLC, Thornwood NY). Serum samples were diluted in phosphate-buffered saline (PBS) solution containing normal goat serum, Tween-20, and powdered nonfat dry milk to block nonspecific antigen binding sites. Sera were first screened at dilutions of 1:16 to 1:64. All sera that are reactive at 1:64 were further tested with two-fold dilutions to 1:8192.

### Study size

An initial power calculation was based on a request for samples from 150 HSA cases from the CCOGC. With 150 HSA cases, assuming the prevalence of *Bartonella* PCR positive cases reported previously in dogs with HSA,[22] using an alpha of 0.05 this study would have 80% power to detect a difference in the prevalence of *Bartonella* spp. DNA in canine cardiac vs. splenic HSA (assuming samples were split evenly between the two tumor locations) if 49% or more, or 7% or less of the cardiac samples were *Bartonella* PCR positive. In addition, assuming the prevalence of each hemotropic pathogen reported previously in dogs with HSA,[22] using an alpha of 0.05 this study would have 80% power to detect similar size differences with 124 cases for hemotropic *Mycoplasma* and 80 cases for *Babesia* spp.

### Statistical methods

Descriptive statistics were obtained for demographic factors (age, weight, sex, breed, season of sample collection, geographic location). We assessed statistical differences in the proportion of dogs PCR positive for each hemotropic pathogen using the Chi-squared test of independence (or Fisher’s exact tests for small sample numbers). We also assessed statistical differences in the proportion of samples PCR positive from each anatomic location using the Chi-squared test of independence (or Fisher’s exact tests for small sample numbers). Differences between demographic factors for *Bartonella* PCR positive and negative dogs were determined using the Wilcoxon Rank-Sum test for continuous variables (age, weight), and Fisher’s exact tests for categorical variables. Multivariable logistic regression was used to identify associations between *Bartonella* infection and anatomic location of tissue sample, with potential demographic confounders included as explanatory variables (age, weight, sex, breed, season of sample collection, and geographic location of sample collection). Odds ratios (adjusted ORs) and 95% confidence intervals (95% CIs) were calculated. To determine agreement between tests on two different samples within the same dog, the kappa statistic was calculated.[39] Data analysis was performed using R 3.6.0 (https://www.R-project.org/).

## Results

**Fig 2:**
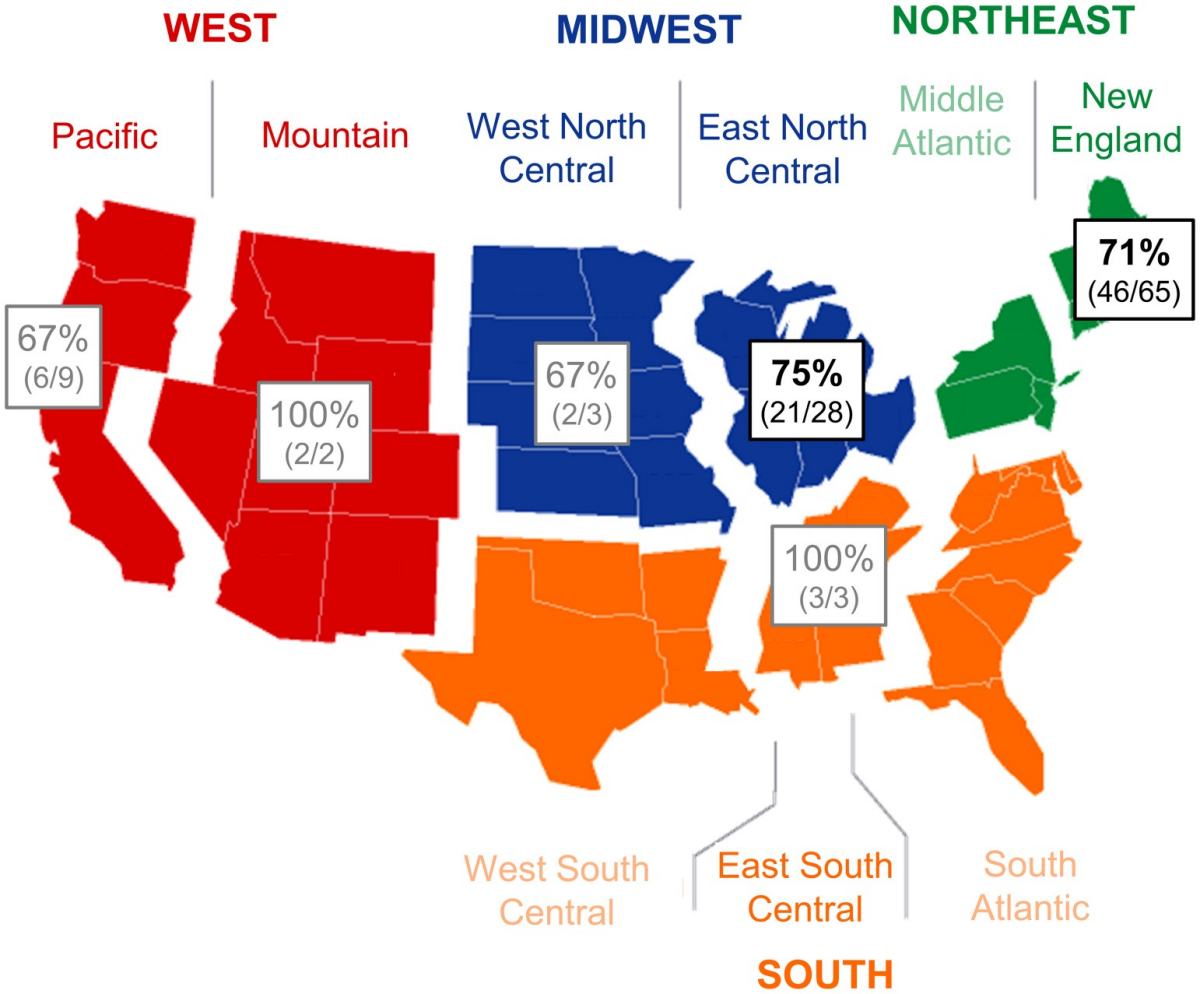
Geographic distribution of dogs with HSA. Shows the percentage of dogs positive for *Bartonella* DNA on PCR of HSA tumor tissue and/or non-tumor tissue biopsy. Color indicates region. Black text indicates regions from which 10 or more samples were received, and gray text indicates regions from < 10 samples were received.

Demographic characteristics of the included dogs are shown in Table 1. There were 58 female dogs (93% spayed) and 52 male dogs (85% neutered). The most common breeds were mixed breed dogs (n = 31), Labrador retrievers (18), and golden retrievers (13); breeds with 3 or fewer individuals were grouped into the Other breed category (34). The median age was 10 years old, and the median weight was 33.2 kg. No weight was provided for 14 dogs. HSA was diagnosed in all four seasons. Most samples were collected at Tufts University (n = 65) and the University of Wisconsin (19), with between 2–9 dogs included from six other participating veterinary schools (Fig 2).

**Table 1:** Demographic and clinicopathologic characteristics of study population. Median and range shown for continuous data. The * indicates a proportion significantly different from baseline (breed baseline = mixed breed).

	**All**		***Bartonella* +**		***Bartonella* -**		***p-value***
	Median	Range	Median	Range	Median	Range	
Age (yrs)	10	4–20	10	4–20	10	6–13	0.438
Weight (kg)	33.2	6.1–68.0	34.0	8.5–68.0	30.3	6.1–48.8	0.800
	**All dogs** Number in each category (%)		**Number *Bartonella* + (%)**				**p-value**
*Sex*							0.31
FI	4 (4)		3 (75)				
FS	54 (49)		37 (69)				
MI	8 (7)		8 (100)				
MN	44 (40)		32 (73)				
*Breed*							0.046
Mix	31 (28)		26 (84)				
Lab	18 (16)		10 (56)*				
Golden	13 (12)		9 (69)				
GSD	6 (5)		2 (33)*				
Boxer	4 (4)		4 (100)				
Bichon Frise	4 (4)		2 (50)				
Other	34 (31)		27 (79)				
*Season*							0.778
Autumn	38 (35)		27 (71)				
Winter	23 (21)		18 (78)				
Spring	20 (18)		13 (65)				
Summer	29 (26)		22 (76)				
*Location*							0.658
MA	65		44 (71)				
WI	19		16 (84)				
CA	9		6 (86)				
MI	6		3 (50)				
MO	3		2 (67)				
OH	3		2 (67)				
TN	3		3 (100)				
CO	2		2 (100)				

The number of samples of each type (fresh frozen HSA tumor tissue, fresh frozen non-tumor tissue, whole blood, serum) provided by the CCOGC is shown in Table 2. Of the 100 dogs with fresh frozen HSA tumor samples submitted, 74 were splenic, 14 were cardiac, and 12 were from other sites (5 liver, 2 SQ, 1 lung, 1 undetermined retroperitoneal, 1 undetermined intrathoracic, and 2 undetermined). Of the 104 dogs with fresh frozen non-tumor tissue samples submitted, 39 were splenic, 14 were cardiac, 49 were skin or various subcutaneous tissues (skin/SQ), and 2 were from other sites (1 liver, 1 kidney). The anatomic location of HSA tumor and non-tumor tissue samples is summarized in Table 2. For dogs with splenic HSA tumors, 43% (32/74) had adjacent non-tumor splenic tissue submitted and 49% (36/74) had skin/SQ non-tumor tissue submitted (the remaining 6 dogs with splenic HSA tumors did not have non-tumor tissue submitted). For dogs with HSA tumors from other anatomic locations (including cardiac), the non-tumor tissues submitted were all from the affected organ.

**Table 2:** Anatomic location of samples. Number of samples tested from each anatomic location, and number of each sample positive for each pathogen tested. Serum was tested for *Bartonella* spp. antibodies using IFA; serology for hemotropic *Mycoplasma* and *Babesia* spp. was not performed. All other samples were tested by PCR for DNA of each pathogen.

	Number of samples	Number of samples (%) *Bartonella* spp. positive	Subgroup of independently confirmed samples: # *Bartonella* spp positive/# tested (% *Bartonella* spp. positive)	Number of samples hemotropic *Mycoplasma* spp. positive	Number of samples *Babesia* spp. positive
*HSA tumors*					
Spleen	74	24 (32)	5/18 (28%)	1	0
Cardiac	14	8 (57)	0/0 (N/A)	0	0
Other	12	2 (17)	0/3 (0%)	0	0
TOTAL	100	34 (34)		1	0
*Non-tumor tissue*					
Spleen	39	22 (56)	19/35 (54%)	0	0
Cardiac	14	13 (93)	0/0 (N/A)	1	0
Skin/SQ	49	31 (63)	31/49 (63%)	1	0
Other	2	0 (0)	0/2 (0%)	0	0
TOTAL	104	66 (63)		2	0
*Blood*					
Whole blood	108	0	NA	2	0
Serum	107	6	NA	N/A	N/A

**Fig 3:**
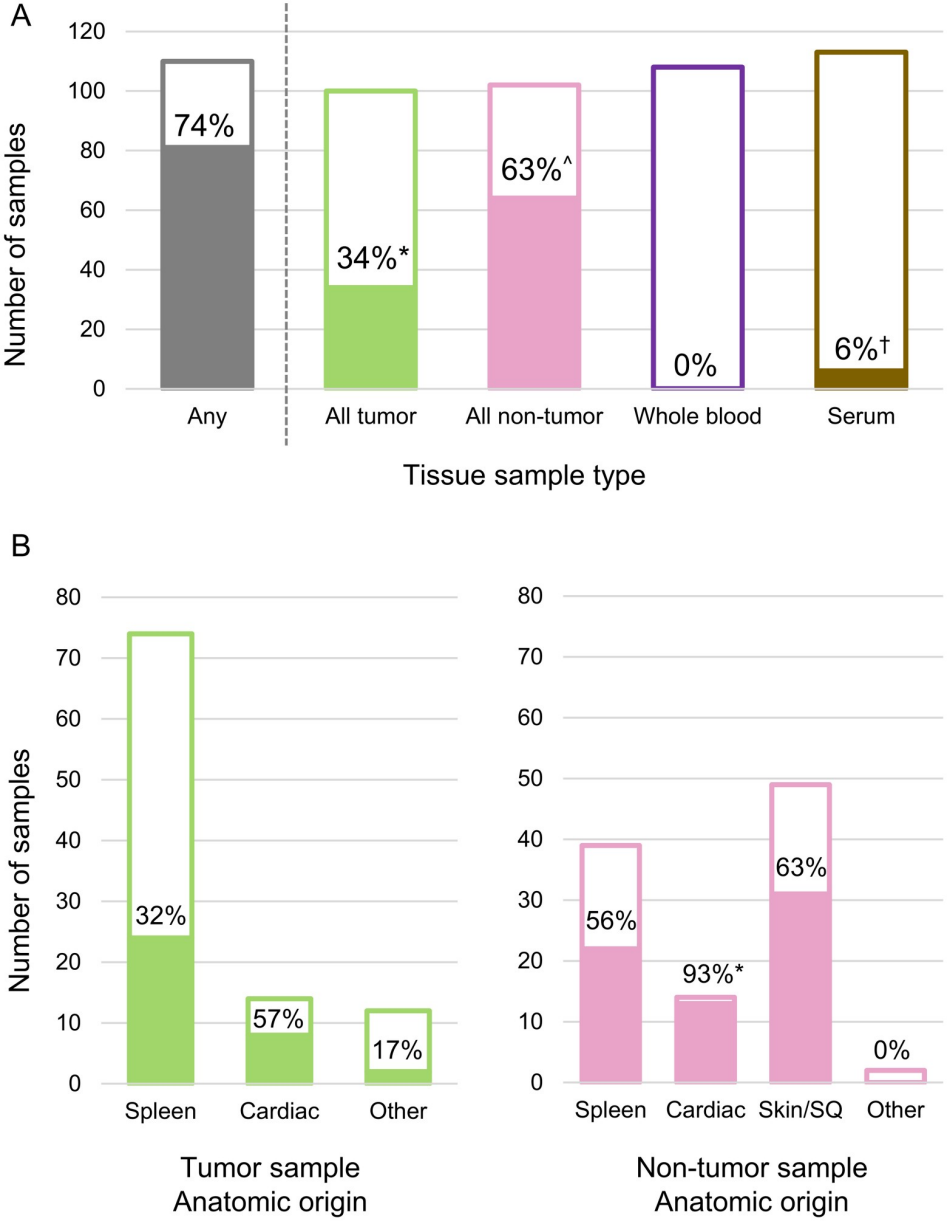
Proportion of samples positive for *Bartonella* spp. Color indicates sample type (HSA tumors, green; non-tumor tissue, pink; whole blood, purple; serum, brown; any sample, grey). A) Blood, serum, HSA tumors, and non-tumor tissues (all anatomic locations combined). Blood and tissue were tested by PCR for *Bartonella* spp. DNA, serum was tested by IFA for *Bartonella* spp. antibodies. Different superscripts indicate significantly different proportions (p < 0.05, Chi-squared test) B) Left panel shows HSA tumors by anatomic location, right panel shows non-tumor tissues by anatomic location. The * indicates a statistically significant difference in proportion from spleen samples (p < 0.05, Chi-squared or Fisher’s exact test).

Hemotropic pathogen testing results for all samples are summarized in Table 2. Of the 110 HSA dogs, 80 (73%) were *Bartonella* spp. PCR positive in at least one fresh frozen tissue sample. No dog was *Bartonella* spp. PCR positive on whole blood and only 6 (6%) were IFA seroreactive to *B*. *henselae*, *B*. *vinsonii* subsp. *berkhoffii*, or *B*. *koehlerae* antigens (Fig 3A). In these 6 dogs, 3 were only seroreactive to *B*. *henselae*, 2 were *B*. *henselae* and *B*. *koehlerae* seroreactive, and 1 was only *B*. *koehlerae* seroreactive.

With the exception of breed, there was no difference in the proportion of dogs *Bartonella* spp. PCR positive based on any of the demographic factors considered (Table 1). The proportion of German Shepherd Dogs (OR 0.60, 95% CI 0.41–0.88) and Labrador retrievers (OR 0.75, 95% CI 0.59–0.97) positive for *Bartonella* spp. DNA was significantly lower than that of mixed breed dogs. When adjusted for other potentially confounding demographic variables using multivariable logistic regression, only Labrador retrievers had a lower proportion positive for *Bartonella* spp. DNA compared to mixed breed dogs (OR 0.69, 95% CI 0.52–0.92). The proportion of *Bartonella* spp. PCR positive dogs from each geographic location, based on the location of the submitting veterinary college, is shown in Fig 2. Tthere were no statistically significant differences in *Bartonella* spp. between geographic locations (Table 1), with *Bartonella* positive dogs distributed broadly throughout the United States.

**Fig 4:**
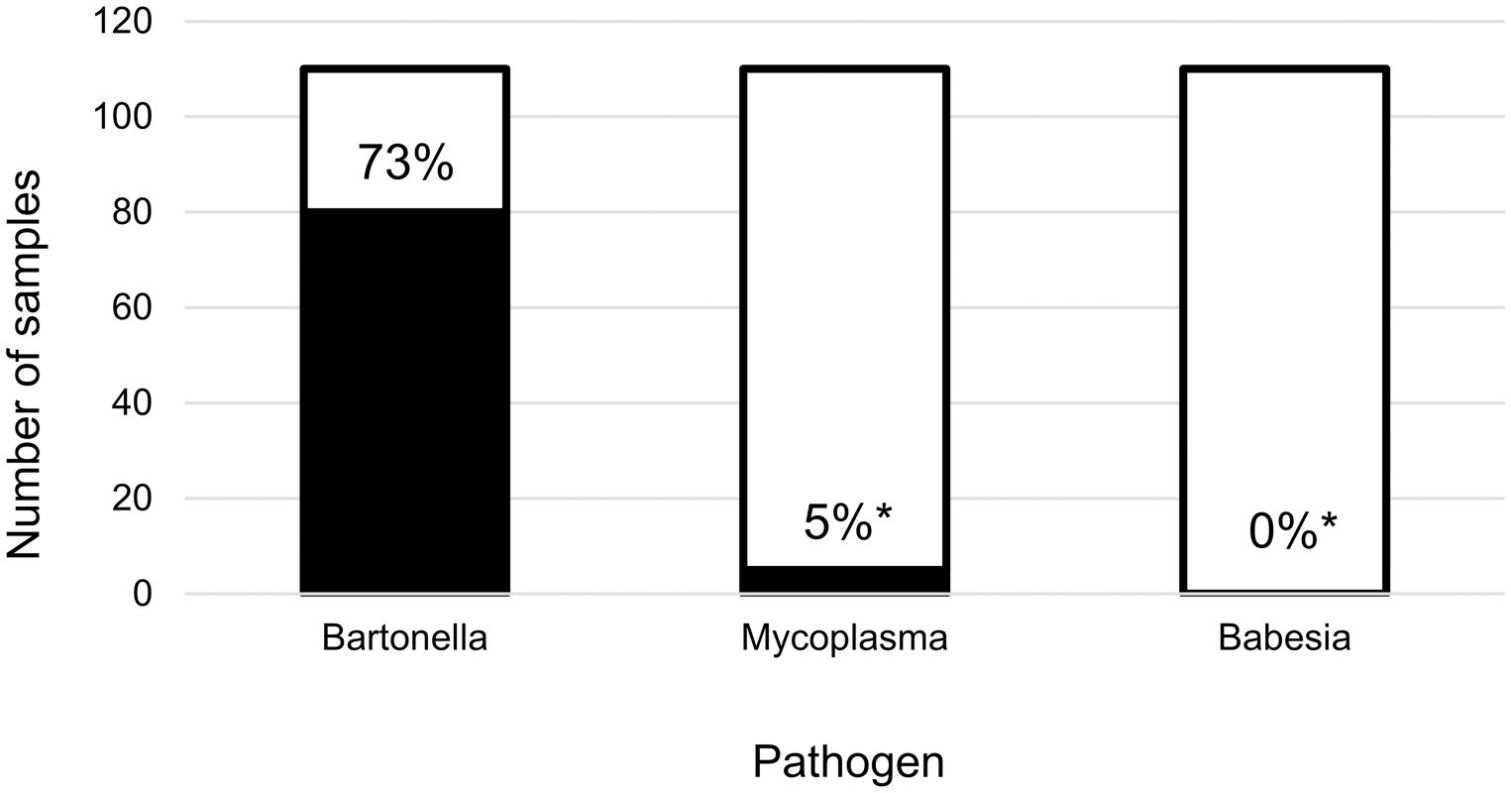
Proportion of dogs with HSA positive for hemotropic pathogen DNA on any sample. * indicates statistically significant difference in proportion from *Bartonella* spp. (p < 0.05, Chi-squared test).

Fresh frozen tissues from only 3 dogs were hemotropic *Mycoplasma* spp. PCR positive, a significantly smaller proportion (3%, p < 0.0001) compared to *Bartonella* spp. (Fig 4). Hemotropic *Mycoplasma* spp. DNA was amplified from two non-tumor tissues (one skin/SQ and one cardiac), and one HSA tumor (spleen). The 2 hemotropic *Mycoplasma* spp. positive non-tumor tissues were both also *Bartonella* spp. PCR positive; the hemotropic *Mycoplasma* positive tumor tissues was *Bartonella* spp. PCR negative. Hemotropic *Mycoplasma* spp. DNA was amplified from whole blood of two dogs, neither of which had hemotropic *Mycoplasma* spp. DNA in their tissues. *Babesia* spp. DNA was not amplified from any whole blood or tissue specimen (Fig 4).

The proportion of HSA tumor and non-tumor tissues *Bartonella* spp. PCR positive for each anatomic location is summarized in Fig 3B. The proportion of non-tumor tissues that were *Bartonella* spp. PCR positive (63%) was significantly higher than the proportion of HSA tumors that were *Bartonella* spp. PCR positive 34%, (p < 0.001). The proportion of *Bartonella* spp. PCR positive HSA tumor samples did not differ significantly between anatomic location (p = 0.083). Based on the multivariable logistic regression model, the anatomic location of the HSA tumor was not associated with *Bartonella* spp. PCR positivity. The proportions of non-tumor tissues *Bartonella* spp. PCR positive were significantly different based on anatomic location (p < 0.0001), with a higher proportion of non-tumor cardiac samples positive for *Bartonella* spp. PCR compared to non-tumor spleen samples when adjusted for possible demographic confounders (adjusted OR 1.75, 95% CI 1.25–2.47).

When comparing the *Bartonella* spp. PCR results for HSA tumor and non-tumor tissues, there was only slight agreement between the two samples (kappa = 0.14). Only 36% of dogs with a *Bartonella* spp. PCR positive non-tumor tissue sample had a positive HSA tumor, whereas 76% of dogs with a positive HSA tumor sample were positive in non-tumor tissue.

In both tumor and non-tumor samples, the most common *Bartonella* species identified was *B*. *henselae*. Homologies ranged from 99.3% to 100% (of 138 bp analyzed) with *B*. *henselae* CAL1 and SA2 (Genbank accessions AF369527and AF369529, respectively). Of the HSA tumors, 27 contained *B*. *henselae* (including 2 co-infected with *B*. *henselae* and *B*. *koehlerae*), 1 contained only *B*. *koehlerae* (140/140 bp, 100% homology with Genbank accession AF312490), 1 contained *Bartonella apis* (94/94 bp, 100% homology with Genbank accession CP015625) and 3 had a *Bartonella* species that was most closely related to *B*. *henselae* (with homology ranging from 95% to 98% with either *B*. *henselae* CAL1 or SA2). In non-tumor tissues, 58 contained *B*. *henselae*, 1 contained *B*. *koehelerae* (140/140 bp, 100% homology with Genbank accession AF312490), 6 contained a *Bartonella* species that was most closely related to *B*. *henselae* (with homology ranging from 95% to 98% with either *B*. *henselae* CAL1 or SA2), and 1 contained a *Bartonella* species that was unable to be identified to the species level.

When considering only those tissue samples that were able to be independently assigned an anatomic location and tumor presence based on histopathology of formalin-fixed samples performed by the investigators, the proportions of each tissue type positive for *Bartonella* spp. DNA by PCR (Table 2) was similar to that of the entire sample set. For HSA tumors in the spleen, the independently confirmed subgroup had 5 of 18 positive (28%), compared to the entire set with 32% positive (p = 0.515). For non-tumor tissues from the spleen, the independently confirmed subgroup had 19 of 35 positive (56%), compared to the entire set with 54% positive (p = 0.960).

## Discussion

Overall, in this study 73% of dogs with HSA were *Bartonella* spp. PCR positive on HSA tumor tissue, non-tumor tissue, or bothtissue samples. Consistent with our previous study,[22] the proportion of *Bartonella* spp. infection in dogs with HSA was significantly greater than the proportion of *Babesia* or hemotropic *Mycoplasma* spp. Somewhat surprisingly, *Bartonella* spp. DNA was amplified more often from non-tumor tissues (63%) compared to HSA tumors tissues (34%).

There are several potential explanations for the difference in *Bartonella* prevalence between HSA tumor and non-tumor tissues. It is possible that in large vascular splenic tumors there are necrotic or infarcted regions that contain fewer organisms, placing these samples below the level of PCR detection. This possibility is supported by amplification of *Bartonella* spp. DNA from 75% of non-tumor tissues obtained from the 32 dogs that were PCR positive from their HSA tumors. It is also possible that the high prevalence of *Bartonella* DNA in non-tumor tissues reflects asymptomatic carriage and is not associated with HSA. However since all 110 dogs had HSA, the higher prevalence in non-tumor tissue does not necessarily contradict the hypothesis that *Bartonella* infection is associated with HSA–rather, in the absence of a control group of dogs without HSA, we cannot conclude whether this high prevalence is seen in the absence of HSA as well. Further studies using a case-control or cohort design to compare *Bartonella* spp. PCR prevalence between dogs with HSA and without HSA will be needed to determine if this is the case.

While the proportion of dogs with *Bartonella* spp. DNA in one or more tissue samples was surprisingly high, no dog had *Bartonella* DNA amplified from a blood sample. This suggests that *Bartonella* DNA found in any given tissue is not due to blood contamination of the tissue, but rather that the *Bartonella* may be intracellularly localized within that tissue. Similar results have been reported previously: in one case dogs experimentally infected with *Bartonella* spp. failed to become detectably bacteremic, despite *Bartonella* spp. being isolated from tissues (bone marrow and lung) post-mortem; in another, a dog had *B*. *henselae* DNA amplified from biopsies of vasculitis lesions and normal skin, but no *Bartonella* DNA on multiple sequential blood samples.[40,41] Reasons that *Bartonella* DNA is not found in blood samples from dogs with *Bartonella* present in tissue could include intermittent bacteremia, rapid clearance of bacteremia by the dogs’ immune system, or low levels of bacteremia that are below the current limit of PCR detection. Regardless, *Bartonella* blood PCR does not effectively predict whether a dog is infected with a *Bartonella* spp. Similarly, *Bartonella* spp. serology was also positive in only a small fraction of those dogs with positive *Bartonella* spp. PCR in tissue. Previous results documenting poor agreement between *Bartonella* spp. exposure based on IFA, and *Bartonella* spp. bacteremia in dogs have been reported.[42,43] Finally, in comparison with FFPE splenic HSA tumors tested for *Bartonella* in our previous study, *Bartonella* testing on fresh-frozen splenic HSA tumors yielded very slightly higher levels of detection (26% of fixed tissue vs. 28–32% of fresh frozen tissue). Based upon this study, we recommend that when possible, fresh frozen tissue biopsies from either the affected organ, or unaffected skin, be collected and submitted for PCR testing to maximize the potential for detection of *Bartonella* spp. Future studies will be needed to guide medical decision making when *Bartonella* spp. infection is documented in a dog with HSA.

To address the possibility that *Bartonella* spp. DNA is found in high proportions of dogs’ spleens due to the spleen’s role in immunologic clearance of bacteria or infected erythrocytes from circulation, we tested multiple other organs for the presence of *Bartonella* DNA. Rather than seeing the highest proportions of splenic tissue positive for *Bartonella* DNA, in both tumor and non-tumor tissues we found cardiac tissues to have a higher proportion positive. This may reflect a tissue tropism of *Bartonella* for cardiac tissue, which would be compatible with the known ability of *Bartonella* to infect heart valves as a cause of endocarditis (or less commonly, myocarditis).[44–47] Additionally, when examining non-tumor tissues, we found a similar proportion of skin/SQ samples were positive for *Bartonella* compared to spleen samples. This may reflect an alternative tissue tropism for the bacteria, which is vector-borne and relies on the bite of an arthropod vector for transmission. Establishing latent infection in the skin could be an evolutionary response to enhance the likelihood of uptake during blood feeding by arthropod vectors. Based on the results presented here, it is unlikely that the high proportion of dog spleen samples positive for *Bartonella* spp. DNA on PCR reflect solely splenic clearance of bacteria or infected erythrocytes.

Because of the surprisingly high proportion of dogs with HSA with *Bartonella* DNA in tissue samples in this study, and the well-established ability of *Bartonella* spp. to induce angiogenesis and chronic inflammation *in vivo* and *in vitro*, we speculate that *B*. *henselae* could be a cause or cofactor in the development of HSA in dogs. Angiogenesis is a fundamental component of primary tumor cell proliferation and metastasis, particularly in HSA.[21,48–52] Specifically, dogs with HSA have increased amounts of plasma VEGF compared to healthy dogs,[48] VEGF and its receptors are present in tumors (when evaluated with IHC)[52] and upregulated in HSA compared to benign hemangioma[49] (and in xenograft tumors using a mouse model)[50]. *In vitro*, *B*. *henselae* induces angiogenesis and proliferation of endothelial cells in part by stimulating production of VEGF.[23–25,31,53,54] It is well established that *Bartonella* spp. cause the non-neoplastic endothelial proliferative disorders bacillary angiomatosis and peliosis hepatis in humans, and there have also been rare reports of these conditions in dogs infected with *Bartonella* spp.[26–31,53,55,56] In addition, there have been case reports documenting neoplastic endothelial cell tumors in humans and dogs infected with *Bartonella* spp.: *B*. *vinsonii* subsp. *berkhoffii* was isolated from a dog with hemangiopericytoma, and from humans residing on three continents with epithelioid hemangioendothelioma.[57,58] The high prevalence of *Bartonella* DNA in tissues from dogs with HSA supports the need for further studies on the mechanistic basis of a potential link between *Bartonella* spp. infection and vascular endothelial cancers like HSA.

Limitations of this study include the use of archived specimens, which precluded systematic sampling from particular organs of interest. This study was designed to be descriptive, and as such there was no control group consisting of dogs without HSA. Because of the lack of control group in this study, we cannot determine whether *Bartonella* spp. infection is epidemiologically associated with HSA. Additionally, only approximately one third of tumor tissue samples were able to be independently reviewed to confirm their anatomic tissue of origin and that they contained neoplastic cells. However, the overall pattern of lower Bartonella DNA prevalence in HSA tumor tissue compared to non-tumor tissue that was evident in both the spleen and cardiac tissues was also present in the subgroup of splenic tissues that did have independent histopathologic confirmation of anatomic location and tumor cell presence. There was not fixed tissue provided for independent histopathologic review from any of the cardiac samples (tumor or non-tumor), so diagnosis relied on the histopathologic reports from the submitting veterinary colleges. This may have led to misclassification of cardiac tissues as tumor or non-tumor. However due to the biological behavior of HSA in the heart, with most tumors presenting as a mass involving the right atrium (even in dogs with concurrent splenic HSA), correct classification of tissue as HSA (mass in right atrium) or non-tumor (other unaffected cardiac tissue) at the time of sample collection was assumed to be accurate. This study was also limited by using IFA to detect *Bartonella* spp. antibodies. IFA is known to have low sensitivity and may underestimate the true seroprevalence of *Bartonella* spp. in dogs.[42,59–62] The use of other serological assays currently under development, such as Western Blot or ELISA, may improve sensitivity of detection of seroreactivity. Finally, the initial power calculations for determining a statistically significant difference in *Bartonella* infection of tumors from different anatomic locations (splenic vs. cardiac) was based on 75 splenic and 75 cardiac cases. In fact, we received many fewer cardiac samples than expected, which decreased the power of this aim of the study. With the sample size used (69 splenic and 13 cardiac), the study was underpowered to detect the empiric difference that was found (33% *Bartonella* PCR positive for splenic and 54% for cardiac). In contrast, because all 105 dogs were able to be tested for each pathogen, despite the slightly smaller sample size than expected this aim was adequately powered to detect the empiric differences found (74% *Bartonella* spp. PCR positive, 3% hemotropic *Mycoplasma* PCR positive, 0% *Babesia* spp. PCR positive).

We conclude that our findings strengthen the need to further investigate the role of *Bartonella* in the development of HSA, particularly with well controlled epidemiologic studies and mechanistic research to identify how this genus may contribute to tumor development. Ultimately, the development of a vaccine to protect dogs against *Bartonella* infection could potentially decrease the prevalence of this highly malignant neoplasm.

## References

[1] Knoll LJ, Hogan DA, Leong JM, Heitman J, Condit RC. Pearls collections: What we can learn about infectious disease and cancer. PLOS Pathog. 2018 3 29;14(3):e1006915 10.1371/journal.ppat.1006915 29596508 PMC5875890

[2] Plummer M, de Martel C, Vignat J, Ferlay J, Bray F, Franceschi S. Global burden of cancers attributable to infections in 2012: a synthetic analysis. Lancet Glob Heal. 2016 9 1;4(9):e609–16. 10.1016/S2214-109X(16)30143-7 27470177

[3] Group IA for R on CW. Biological Agents: A review of human carcinogens. Vol. 100B, IARC Monographs on the Evaluation of Carcinogenic Risks to Humans. Geneva, Switzerland; 2012.

[4] Riley DR, Sieber KB, Robinson KM, White JR, Ganesan A, Nourbakhsh S, et al Bacteria-Human Somatic Cell Lateral Gene Transfer Is Enriched in Cancer Samples. PLoS Comput Biol. 2013;9(6). 10.1371/journal.pcbi.1003107 PMC3688693 23840181

[5] Lax AJ, Thomas W, Kuper H, Parsonnet J, et al How bacteria could cause cancer: one step at a time. Trends Microbiol. 2002 6;10(6):293–9. 10.1016/s0966-842x(02)02360-0 12088666

[6] Molnar B, Galamb O, Sipos F, Leiszter K, Tulassay Z. Molecular Pathogenesis of Helicobacter pylori Infection: The Role of Bacterial Virulence Factors. Dig Dis. 2010;28(4–5):604–8. 10.1159/000320060 21088410

[7] Pagano JS, Blaser M, Buendia M-A, Damania B, Khalili K, Raab-Traub N, et al Infectious agents and cancer: criteria for a causal relation. Semin Cancer Biol. 2004 12;14(6):453–71. 10.1016/j.semcancer.2004.06.009 15489139

[8] Prymak C, McKee LJ, Goldschmidt MH, Glickman LT. Epidemiologic, clinical, pathologic, and prognostic characteristics of splenic hemangiosarcoma and splenic hematoma in dogs: 217 cases (1985). J Am Vet Med Assoc. 1988 9 15;193(6):706–12. 3192450

[9] Mullin C, Clifford CA. Histiocytic Sarcoma and Hemangiosarcoma Update. Vet Clin North Am Small Anim Pract. 2019 6 8; 10.1016/j.cvsm.2019.04.009 31186126

[10] Spangler WL, Culbertson MR. Prevalence, type, and importance of splenic diseases in dogs: 1,480 cases (1985–1989). J Am Vet Med Assoc. 1992 3 15;200(6):829–34. 1568933

[11] Schultheiss PC. A Retrospective Study of Visceral and Nonvisceral Hemangiosarcoma and Hemangiomas in Domestic Animals. J Vet Diagnostic Investig. 2004 11 25;16(6):522–6. 10.1177/104063870401600606 15586567

[12] Grüntzig K, Graf R, Boo G, Guscetti F, Hässig M, Axhausen KW, et al Swiss Canine Cancer Registry 1955–2008: Occurrence of the Most Common Tumour Diagnoses and Influence of Age, Breed, Body Size, Sex and Neutering Status on Tumour Development. J Comp Pathol. 2016 8;155(2–3):156–70. 10.1016/j.jcpa.2016.05.011 27406312

[13] Hart BL, Hart LA, Thigpen AP, Willits NH. Neutering of German Shepherd Dogs: associated joint disorders, cancers and urinary incontinence. Vet Med Sci. 2016 8;2(3):191–9. 10.1002/vms3.34 29067194 PMC5645870

[14] Kent MS, Burton JH, Dank G, Bannasch DL, Rebhun RB. Association of cancer-related mortality, age and gonadectomy in golden retriever dogs at a veterinary academic center (1989–2016). Bauer JA, editor. PLoS One. 2018 2 6;13(2):e0192578 10.1371/journal.pone.0192578 29408871 PMC5800597

[15] Treggiari E, Pedro B, Dukes-McEwan J, Gelzer AR, Blackwood L. A descriptive review of cardiac tumours in dogs and cats. Vet Comp Oncol. 2017 6;15(2):273–88. 10.1111/vco.12167 26420436

[16] Dana M. ONLINE CASE REPORTS Pericardial Hemangiosarcoma in a 10-Year-Old Papillon.

[17] Johnson KA, Powers BE, Withrow SJ, Sheetz MJ, Curtis CR, Wrigley RH. Splenomegaly in dogs. Predictors of neoplasia and survival after splenectomy. J Vet Intern Med. 3(3):160–6. 10.1111/j.1939-1676.1989.tb03092.x 2778749

[18] Schick AR, Hayes GM, Singh A, Mathews KG, Higginbotham ML, Sherwood JM. Development and validation of a hemangiosarcoma likelihood prediction model in dogs presenting with spontaneous hemoabdomen: The HeLP score. J Vet Emerg Crit Care. 2019 5 17;29(3):239–45. 10.1111/vec.12838 30994972

[19] Batschinski K, Nobre A, Vargas-Mendez E, Tedardi M V, Cirillo J, Cestari G, et al Canine visceral hemangiosarcoma treated with surgery alone or surgery and doxorubicin: 37 cases (2005–2014). Can Vet J = La Rev Vet Can. 2018;59(9):967–72. PMC6091137 30197439

[20] Moore AS, Rassnick KM, Frimberger AE. Evaluation of clinical and histologic factors associated with survival time in dogs with stage II splenic hemangiosarcoma treated by splenectomy and adjuvant chemotherapy: 30 cases (2011–2014). J Am Vet Med Assoc. 2017 9 1;251(5):559–65. 10.2460/javma.251.5.559 28828962

[21] Tamburini BA, Phang TL, Fosmire SP, Scott MC, Trapp SC, Duckett MM, et al Gene expression profiling identifies inflammation and angiogenesis as distinguishing features of canine hemangiosarcoma. BMC Cancer. 2010 12 9;10(1):619. 21062482 10.1186/1471-2407-10-619 PMC2994824

[22] Varanat M, Maggi RG, Linder KE, Breitschwerdt EB. Molecular Prevalence of Bartonella, Babesia, and Hemotropic Mycoplasma sp. in Dogs with Splenic Disease. J Vet Intern Med. 2011;25(6):1284–91. 10.1111/j.1939-1676.2011.00811.x 22092618

[23] Beerlage C, Varanat M, Linder K, Maggi RG, Cooley J, Kempf VAJ, et al Bartonella vinsonii subsp. berkhoffii and Bartonella henselae as potential causes of proliferative vascular diseases in animals. Med Microbiol Immunol. 2012 8 27;201(3):319–26. 10.1007/s00430-012-0234-5 22450733

[24] Kempf VAJ, Volkmann B, Schaller M, Sander CA, Alitalo K, Rieß T, et al Evidence of a leading role for VEGF in *Bartonella henselae* -induced endothelial cell proliferations. Cell Microbiol. 2001 9;3(9):623–32. 10.1046/j.1462-5822.2001.00144.x 11553014

[25] Devraj G, Beerlage C, Brüne B, Kempf VAJ. Hypoxia and HIF-1 activation in bacterial infections. Microbes Infect. 2017 3;19(3):144–56. 10.1016/j.micinf.2016.11.003 27903434

[26] Berkowitz ST, Gannon KM, Carberry CA, Cortes Y. Resolution of spontaneous hemoabdomen secondary to peliosis hepatis following surgery and azithromycin treatment in a Bartonella species infected dog. J Vet Emerg Crit Care. 2016;00(0):1–7. 10.1111/vec.12479 27074964

[27] Yager JA, Best SJ, Maggi RG, Varanat M, Znajda N, Breitschwerdt EB. Bacillary angiomatosis in an immunosuppressed dog. Vet Dermatol. 2010 3;21(4):420–8. 10.1111/j.1365-3164.2010.00879.x 20374571

[28] Kostianovsky M. Angiogenic process in bacillary angiomatosis. Ultrastruct Pathol. 1994;18(3):349 10.3109/01913129409023203 7520641

[29] Perkocha LA, Geaghan SM, Yen TS, Nishimura SL, Chan SP, Garcia-Kennedy R, et al Clinical and pathological features of bacillary peliosis hepatis in association with human immunodeficiency virus infection. Vol. 323, The New England journal of medicine. 1990 p. 1581–6. 10.1056/NEJM199012063232302 2233946

[30] Anstead GM. The centenary of the discovery of trench fever, an emerging infectious disease of World War 1. Lancet Infect Dis. 2016;16(8):e164–72. 10.1016/S1473-3099(16)30003-2 27375211 PMC7106389

[31] Berrich M, Kieda C, Grillon C, Monteil M, Lamerant N, Gavard J, et al Differential effects of bartonella henselae on human and feline macro- and micro-vascular endothelial cells. PLoS One. 2011;6(5). 10.1371/journal.pone.0020204 PMC3103534 21637717

[32] Lashnits E, Correa M, Hegarty BC, Birkenheuer A, Breitschwerdt EB. Bartonella Seroepidemiology in Dogs from North America, 2008–2014. J Vet Intern Med. 2018 1;32(1):222–31. 10.1111/jvim.14890 29197186 PMC5787158

[33] Yancey CB, Hegarty BC, Qurollo BA, Levy MG, Birkenheuer AJ, Weber DJ, et al Regional seroreactivity and vector-borne disease co-exposures in dogs in the United States from 2004–2010: utility of canine surveillance. Vector Borne Zoonotic Dis. 2014;14(10):724–32. 10.1089/vbz.2014.1592 25325316

[34] Mazcko C, Thomas R. The Establishment of the Pfizer-Canine Comparative Oncology and Genomics Consortium Biospecimen Repository. Vet Sci. 2015 7 7;2(3):127–30. 10.3390/vetsci2030127 29061936 PMC5644634

[35] Breitschwerdt EB, Maggi RG, Duncan AW, Nicholson WL, Hegarty BC, Woods CW. Bartonella Species in Blood of Immunocompetent Persons with Animal and Arthropod Contact. Emerg Infect Dis. 2007 6;13(6):938–41. 10.3201/eid1306.061337 17553243 PMC2792845

[36] Varanat M, Maggi RG, Linder KE, Horton S, Breitschwerdt EB. Cross-contamination in the Molecular Detection of *Bartonella* from Paraffin-embedded Tissues. Vet Pathol. 2009 9 9;46(5):940–4. 10.1354/vp.08-VP-0259-B-BC 19429988

[37] Maggi RG, Birkenheuer AJ, Hegarty BC, Bradley JM, Levy MG, Breitschwerdt EB. Comparison of serological and molecular panels for diagnosis of vector-borne diseases in dogs. Parasit Vectors. 2014;7(1):127. 24670154 10.1186/1756-3305-7-127 PMC3972965

[38] Maggi RG, Mascarelli PE, Pultorak EL, Hegarty BC, Bradley JM, Mozayeni BR, et al Bartonella spp. bacteremia in high-risk immunocompetent patients. Diagn Microbiol Infect Dis. 2011;71(4):430–7. 10.1016/j.diagmicrobio.2011.09.001 21996096

[39] McHugh ML. Interrater reliability: the kappa statistic. Biochem medica. 2012;22(3):276–82. PMC3900052 23092060

[40] Balakrishnan N, Cherry Na, Linder KE, Pierce E, Sontakke N, Hegarty BC, et al Experimental infection of dogs with Bartonella henselae and Bartonella vinsonii subsp. berkhoffii. Vet Immunol Immunopathol. 2013;156(1–2):153–8. 10.1016/j.vetimm.2013.09.007 24120155

[41] Southern BL, Neupane P, Ericson ME, Dencklau JC, Linder KE, Bradley JM, et al *Bartonella henselae* in a dog with ear tip vasculitis. Vet Dermatol. 2018 12;29(6):537–e180. 10.1111/vde.12695 30318847

[42] Neupane P, Hegarty BC, Marr HS, Maggi RG, Birkenheuer AJ, Breitschwerdt EB. Evaluation of cell culture-grown *Bartonella* antigens in immunofluorescent antibody assays for the serological diagnosis of bartonellosis in dogs. J Vet Intern Med. 2018 11 1;32(6):1958–64. 10.1111/jvim.15301 30307643 PMC6271329

[43] Pérez Vera C, Diniz PPVP, Pultorak EL, Maggi RG, Breitschwerdt EB. An unmatched case controlled study of clinicopathologic abnormalities in dogs with Bartonella infection. Comp Immunol Microbiol Infect Dis. 2013;36(5):481–7. 10.1016/j.cimid.2013.04.001 23683861

[44] Chomel BB, Kasten RW, Williams C, Wey AC, Henn JB, Maggi R, et al Bartonella endocarditis: a pathology shared by animal reservoirs and patients. Ann N Y Acad Sci. 2009;1166:120–6. 10.1111/j.1749-6632.2009.04523.x 19538271

[45] MacDonald KA, Chomel BB, Kittleson MD, Kasten RW, Thomas WP, Pesavento P. A Prospective Study of Canine Infective Endocarditis in Northern California (1999–2001): Emergence of Bartonella as a Prevalent Etiologic Agent. J Vet Intern Med. 2004 1;18(1):56–64. 10.1892/0891-6640(2004)18<56:apsoci>2.0.co;2 14765733

[46] Roura X, Santamarina G, Tabar M-D, Francino O, Altet L. Polymerase chain reaction detection of Bartonella spp. in dogs from Spain with blood culture-negative infectious endocarditis. J Vet Cardiol. 2018 8 25;20(4):267–75. 10.1016/j.jvc.2018.04.006 29807750

[47] Fenimore A, Varanat M, Maggi R, Schultheiss P, Breitschwerdt E, Lappin MR. Bartonella spp. DNA in cardiac tissues from dogs in colorado and wyoming. J Vet Intern Med. 2011 5;25(3):613–6. 10.1111/j.1939-1676.2011.0722.x 21539606

[48] Clifford CA, Hughes D, Beal MW, Mackin AJ, Henry CJ, Shofer FS, et al Plasma Vascular Endothelial Growth Factor Concentrations in Healthy Dogs and Dogs with Hemangiosarcoma. J Vet Intern Med. 2001;15(2):131 10.1892/0891-6640(2001)015<0131:pvegfc>2.3.co;2 11300596

[49] Yonemaru K, Sakai H, Murakami M, Yanai T, Masegi T. Expression of vascular endothelial growth factor, basic fibroblast growth factor, and their receptors (flt-1, flk-1, and flg-1) in canine vascular tumors. Vet Pathol. 2006;43(6):971–80. 10.1354/vp.43-6-971 17099154

[50] Kodama A, Sakai H, Matsuura S, Murakami M, Murai A, Mori T, et al Establishment of canine hemangiosarcoma xenograft models expressing endothelial growth factors, their receptors, and angiogenesis-associated homeobox genes. BMC Cancer. 2009 10 14;9:363 10.1186/1471-2407-9-363 19825192 PMC2768746

[51] Abou Asa S, Mori T, Maruo K, Khater A, El-sawak A, Abd el-Aziz E, et al Analysis of genomic mutation and immunohistochemistry of platelet-derived growth factor receptors in canine vascular tumours. Vet Comp Oncol. 2015 9 1;13(3):237–45. 10.1111/vco.12035 23611531

[52] Göritz M, Müller K, Krastel D, Staudacher G, Schmidt P, Kühn M, et al Canine splenic haemangiosarcoma: Influence of metastases, chemotherapy and growth pattern on post-splenectomy survival and expression of angiogenic factors. J Comp Pathol. 2013 7;149(1):30–9. 10.1016/j.jcpa.2012.11.234 23276383

[53] Pons MJ, Gomes C, Aguilar R, Barrios D, Aguilar-Luis MA, Ruiz J, et al Immunosuppressive and angiogenic cytokine profile associated with Bartonella bacilliformis infection in post-outbreak and endemic areas of Carrion’s disease in Peru. Caimano MJ, editor. PLoS Negl Trop Dis. 2017 6 19;11(6):e0005684 10.1371/journal.pntd.0005684 28628613 PMC5491314

[54] Scheidegger F, Quebatte M, Mistl C, Dehio C. The Bartonella henselae VirB/Bep system interferes with vascular endothelial growth factor (VEGF) signalling in human vascular endothelial cells. Cell Microbiol. 2011 3 1;13(3):419–31. 10.1111/j.1462-5822.2010.01545.x 21044238

[55] Cerimele F, Brown LF, Bravo F, Ihler GM, Kouadio P, Arbiser JL. Infectious Angiogenesis: Bartonella bacilliformis Infection Results in Endothelial Production of Angiopoetin-2 and Epidermal Production of Vascular Endothelial Growth Factor. Am J Pathol. 2003 10 1;163(4):1321–7. 10.1016/S0002-9440(10)63491-8 14507641 PMC1868281

[56] Kitchell BE, Fan TM, Kordick D, Breitschwerdt EB, Wollenberg G, Lichtensteiger CA. Peliosis hepatis in a dog infected with Bartonella henselae. J Am Vet Med Assoc. 2000 2;216(4):519–23. 10.2460/javma.2000.216.519 10687006

[57] Breitschwerdt EB, Maggi RG, Varanat M, Linder KE, Weinberg G. Isolation of Bartonella vinsonii subsp. berkhoffii genotype II from a boy with epithelioid hemangioendothelioma and a dog with hemangiopericytoma. J Clin Microbiol. 2009 6;47(6):1957–60. 10.1128/JCM.00069-09 19369441 PMC2691088

[58] Mascarelli PE, Iredell JR, Maggi RG, Weinberg G, Breitschwerdt EB. Bartonella species bacteremia in two patients with epithelioid hemangioendothelioma. J Clin Microbiol. 2011 11 1;49(11):4006–12. 10.1128/JCM.05527-11 21918021 PMC3209129

[59] Sykes JE, Westropp JL, Kasten RW, Chomel BB. Association between Bartonella species infection and disease in pet cats as determined using serology and culture. J Feline Med Surg. 2010; 10.1016/j.jfms.2010.04.003 PMC10911486 20570199

[60] Brenner EC, Chomel BB, Singhasivanon O-U, Namekata DY, Kasten RW, Kass PH, et al Bartonella infection in urban and rural dogs from the tropics: Brazil, Colombia, Sri Lanka and Vietnam. Epidemiol Infect. 2012;141(1):1–8. 22459880 10.1017/S0950268812000519 PMC9152078

[61] Cherry N, Diniz P, Maggi R, Hummel J, Hardie E, Behrend E, et al Isolation or Molecular Detection of Bartonella henselae and Bartonella vinsonii subsp. berkhoffii from Dogs with Idiopathic Cavitary Effusions. J Vet Intern Med. 2009;23(1):186–9. 10.1111/j.1939-1676.2008.0246.x 19175739

[62] Perez C, Maggi RG, Diniz PPVP, Breitschwerdt EB. Molecular and serological diagnosis of Bartonella infection in 61 dogs from the United States. J Vet Intern Med. 2011;25(4):805–10. 10.1111/j.1939-1676.2011.0736.x 21615498

